# 基于金属有机骨架材料固定相的气相色谱分离应用

**DOI:** 10.3724/SP.J.1123.2020.06028

**Published:** 2021-01-08

**Authors:** Wenqi TANG, Shasha MENG, Ming XU, Zhiyuan GU

**Affiliations:** 南京师范大学化学与材料科学学院, 江苏 南京 210023; School of Chemistry and Materials Science, Nanjing Normal University, Nanjing 210023, China; 南京师范大学化学与材料科学学院, 江苏 南京 210023; School of Chemistry and Materials Science, Nanjing Normal University, Nanjing 210023, China; 南京师范大学化学与材料科学学院, 江苏 南京 210023; School of Chemistry and Materials Science, Nanjing Normal University, Nanjing 210023, China; 南京师范大学化学与材料科学学院, 江苏 南京 210023; School of Chemistry and Materials Science, Nanjing Normal University, Nanjing 210023, China

**Keywords:** 气相色谱, 固定相, 手性分离, 金属有机骨架, gas chromatography (GC), stationary phase, chiral separation, metal-organic frameworks (MOFs)

## Abstract

金属有机骨架材料(MOFs)是一类由有机配体和金属离子(或金属簇)自组装形成的新型多功能材料。MOFs具有孔隙度高、比表面积大、孔径可调、化学和热稳定性高等特点,被广泛应用于吸附、分离、催化等多个领域。近年来,MOFs作为新型气相色谱固定相用于分离异构体受到了广泛关注。与传统无机多孔材料相比,MOFs在结构和功能上展现出高度的可调性,通过合理地选择配体和金属中心,可以设计合成具有不同孔道大小和孔道环境的MOFs,从而分别从热力学和动力学角度优化色谱分离效果,有效提高分离选择性。该文结合MOFs的结构,讨论了MOFs气相色谱固定相分离不同类型分析物的分离机理。分离机理主要包括MOFs孔道的分子筛效应或形状选择性,MOFs不饱和的金属位点与分析物中不同的官能团之间产生的相互作用,分析物与MOFs孔道之间产生的不同范德华力、*π-π*相互作用和氢键相互作用。此外,MOFs的手性分离可能主要依赖于外消旋体与手性MOFs中手性活性位点之间的相互作用。该文也对不同分析目标物进行了归类,综述了多种MOFs气相色谱固定相对烷烃、二甲苯异构体和乙基甲苯、外消旋体、含氧有机物、环境有机污染物的气相色谱分离效果。最后,该文还对MOFs在该领域的应用进行了总结与展望,旨在为MOFs气相色谱高效分离的研究提供参考。

## Abstract (en)

Metal-organic frameworks (MOFs) are a new class of porous materials, which are synthesized using organic ligands and inorganic metal ions or metal clusters. MOFs possess tunable structures through the self-assembly of a large number of organic linkers and metal nodes, which is beyond the scope of conventional porous materials. In addition, MOFs have excellent properties, including the lowest density (as low as 0.13 g/cm), highest specific surface area (as high as 10400 m^2^/g), and largest pore aperture (as large as 9.8 nm) among all porous materials reported till date. Because of their high porosity, large surface area, tunable apertures, as well as high chemical and thermal stabilities, MOFs have been widely applied in the fields of adsorption, separation, and catalysis. In addition, MOFs have been successfully applied as stationary phases for isomer separation in gas chromatography (GC). Since the use of the first MOF (MOF-508) packed column for the separation of alkane isomers in GC, several other MOFs (e. g., MIL-47, MOF-5, and ZIF-8) have been employed for the GC separation of isomers. However, packed-column-type separation not only requires gram-scale quantities of MOFs, thereby increasing the analysis cost, but also results in poor separation efficiency. The first MOF (MIL-101) capillary column designed toward cost reduction allowed for the baseline separation of xylene and ethylbenzene isomers within 100 s under constant-temperature conditions. Since then, the capillary-type column has been widely utilized in the MOF-based stationary phase for GC separation.

Alkanes, xylene isomers and ethyl toluene, oxy-organics and organic pollutants are not only important chemicals in industry but also harmful environmental pollutants. Thus, the separation of these analytes is of practical importance environmental monitoring and industrial quality control. However, it is difficult to realize the efficient separation and detection of these isomers or racemates because of their similar boiling points and molecular sizes. In the past decades, GC was utilized as a rapid and efficient technique for the separation of the abovementioned analytes. The stationary phase used in GC plays a dominant role in the separation processes. This review summarizes the MOF-based GC separation of the abovementioned targets based on the different classification of analytes, including alkanes, xylenes, racemates, oxy-organics and persistent organic pollutants.

The separation mechanisms of different analytes are also discussed according to the structural benefits of MOFs. The separation mechanisms mainly involve van der Waals forces between the MOFs and analytes, interactions between the unsaturated metal sites and different functional groups of the analytes, molecular sieve effect or shape selectivity, and hydrogen-bond or *π-π* interactions. In addition, the chiral recognition abilities of MOFs possibly depend on the interactions between the chiral active sites in chiral MOFs and racemates.

Furthermore, efficient GC separation is influenced by thermodynamic and kinetic factors. The thermodynamic factor is mainly the difference between the partition coefficients of the separated components, which also reflects the properties of the analytes as well as the interactions between the stationary phase and the analytes. The kinetic factor also affects the column efficiency and chromatographic peak shape. Compared with traditional inorganic porous materials, MOFs with tunable structures are more favorable for optimizing the separation of isomers from both thermodynamic and kinetic standpoints. Therefore, this review summarizes the separation mechanism when using MOFs as stationary phases for isomer separation via thermodynamic and kinetic analyses. We hope the review would aid the state-of-art design of MOF stationary phases for high efficient isomer separations in GC.

金属有机骨架材料(MOFs)是一类由有机配体和无机金属离子(或金属簇)自组装形成的高度有序的多孔框架材料^[1]^,因其结构多样、孔道规整可调和孔隙率高,在分离^[2,3,4]^、吸附^[5,6,7]^、传感^[8,9,10]^和催化^[11,12,13]^等领域有着广泛的应用。与传统的无机多孔沸石材料相比,MOFs在结构和功能上展现出高度的可调性。通过选择不同的有机配体和金属中心,可以合成具有不同孔尺寸和结构的多功能MOFs材料。例如,在具有相同拓扑结构的MOFs材料中,引入不同的基团对其孔道进行修饰,使具有相同拓扑结构的材料获得不同的孔道环境,从而展现出不同的分离选择性^[14]^。与此同时,MOFs具有精确到原子级别的结构信息,可以从分子水平上对分离空腔进行设计,使其与目标物匹配,这是大多数传统多孔材料无法达到的。例如,修饰了手性基团的MOFs气相色谱固定相可以进行高效的手性拆分。

近年来,具有不同孔尺寸的MOFs固定相成功应用于不同的色谱法中,例如高效液相色谱法(HPLC)^[15,16,17]^、毛细管电泳色谱法(CEC)^[18,19,20]^和气相色谱法(GC)^[21,22,23,24,25,26]^。与其他色谱法相比,气相色谱法对挥发性较强的小分子具有较高的分辨率、灵敏度和重现性。MOFs固定相可涂覆在毛细管柱内壁上或制备成填充柱作为气相色谱的高效分离介质(见图1)。本文归纳了MOFs固定相分离不同种类目标物的例子,例如烷烃、二甲苯、外消旋体、含氧有机物和环境有机污染物。此外,本文还比较了MOFs和传统吸附型固定相在色谱分离中的性能差异。

**图 1:**
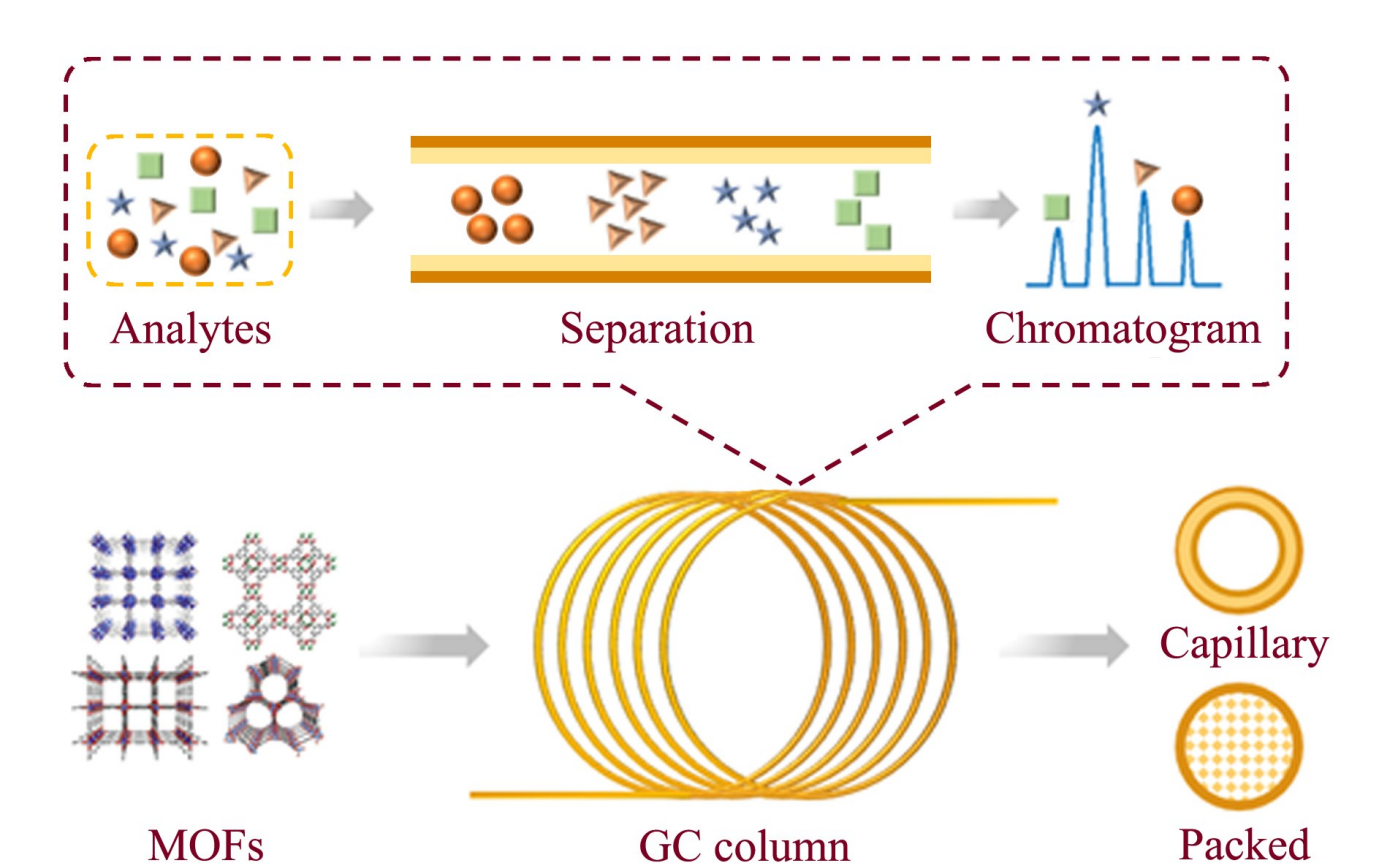
MOFs作为固定相用于气相色谱分离示意图

气相色谱法也是一种研究多孔固体吸附剂分离性能与机理的分析手段。首先,气相色谱固定相涂覆于毛细管内壁通常只需消耗几毫克材料,研究成本低。其次,气相色谱法在研究固定相与分析物之间的相互作用时与液相色谱法不同,不受流动相溶剂的干扰,其分离结果直接体现固定相与分析物之间的相互作用强弱。因此,采用气相色谱法研究MOFs的分离机理具有明显优势。近年来,MOFs应用于气相色谱分离的例子已有报道^[27,28,29,30]^,本文结合MOFs的性质和结构特点,讨论了MOFs气相色谱固定相分离不同类型分析物的优缺点和分离机理。

## 1 MOFs气相色谱固定相的定向设计

传统的气液色谱固定相主要包括聚硅氧烷、聚乙二醇和环糊精,气固色谱固定相包括三氧化二铝、分子筛、活性炭等,它们在化工生产、环境分析和食品安全检测等领域得到了广泛的应用。然而聚合物类固定相的活性位点暴露于水分和氧气中,会引起固定相的降解,易造成固定相流失。因此要不断开发具有低流失、耐高温、分离选择性好的色谱固定相。新型多孔材料MOFs不仅比表面积大、孔隙大小可调、易修饰,而且部分MOFs具有优良的热稳定性和化学稳定性,其作为固定相提供了良好的稳定性、柱寿命和选择性。

气相色谱的分离效果主要受热力学和动力学因素的影响。其中,热力学因素主要由固定相和分析物本身的性质决定。MOFs结构和功能具有高度的可调性,通过对其结构进行调控,有利于从热力学角度优化色谱分离效果,具体调控方式可分为3种:首先,更换不同的有机配体和金属中心,可以设计合成与分析物形状和尺寸高度匹配度的MOFs固定相,实现对目标物的高选择性。其次,利用MOFs中的不饱和金属位点,作为独特的路易斯酸位点,调控金属中心的不饱和金属位点可以与碳-碳双键或三键配位产生特殊的相互作用,从而提高材料的吸附选择性。最后,调控二维MOFs纳米片之间的堆积方式,不同堆积方式的二维MOFs固定相提供不同的分离孔道环境,通过调控其规整或扭曲堆叠可提高对不同分析物的选择性。

动力学因素主要影响峰形和色谱柱的柱效,根据范蒂姆特方程:


(1) $$H=\frac{2D_{g}}{u}+\left[\frac{1+6k+11k^{2}}{24{\left(1+k\right)}^{2}}\times \frac{r^{2}}{D_{g}}+\right.\left.\frac{k}{6{\left(1+k\right)}^{2}}\times \frac{{d_{f}}^{2}}{D_{l}\beta^{2}}\right]\mathrm{\times u}$$

式(1)中,*H*为理论塔板高度,*D*_g_和*D*_l_分别为组分在气相和液相(固定相)中的扩散系数,*u*为线流速,*k*为保留因子,*r*为毛细管半径,*d*_f_为固定相厚度,*β*为相比率。减小固定相中的传质阻力系数,可提高柱效,改善色谱分离效果。减小MOFs固定相传质系数主要有两种途径:一方面,增大固定相扩散系数。例如MIL-101中既保留了UiO-66分离二甲苯异构体和乙基苯所需的正四面体孔,又提供了适量的介孔,有效提高了分析物在MOFs固定相中的扩散效率,实现了100 s内基线分离4种异构体(见图2)^[31,32]^。另一方面,减小固定相的厚度。通过自下而上或自上而下的方法合成具有超薄厚度的新型二维MOFs材料,可有效减小固定相厚度。例如,报道的三维Zr-BTB作为固定相无分离异构体的能力,而具有更薄厚度的二维Zr-BTB纳米片能有效分离二甲苯、二氯苯、乙基甲苯等6组二取代苯异构体^[33]^。

**图 2:**
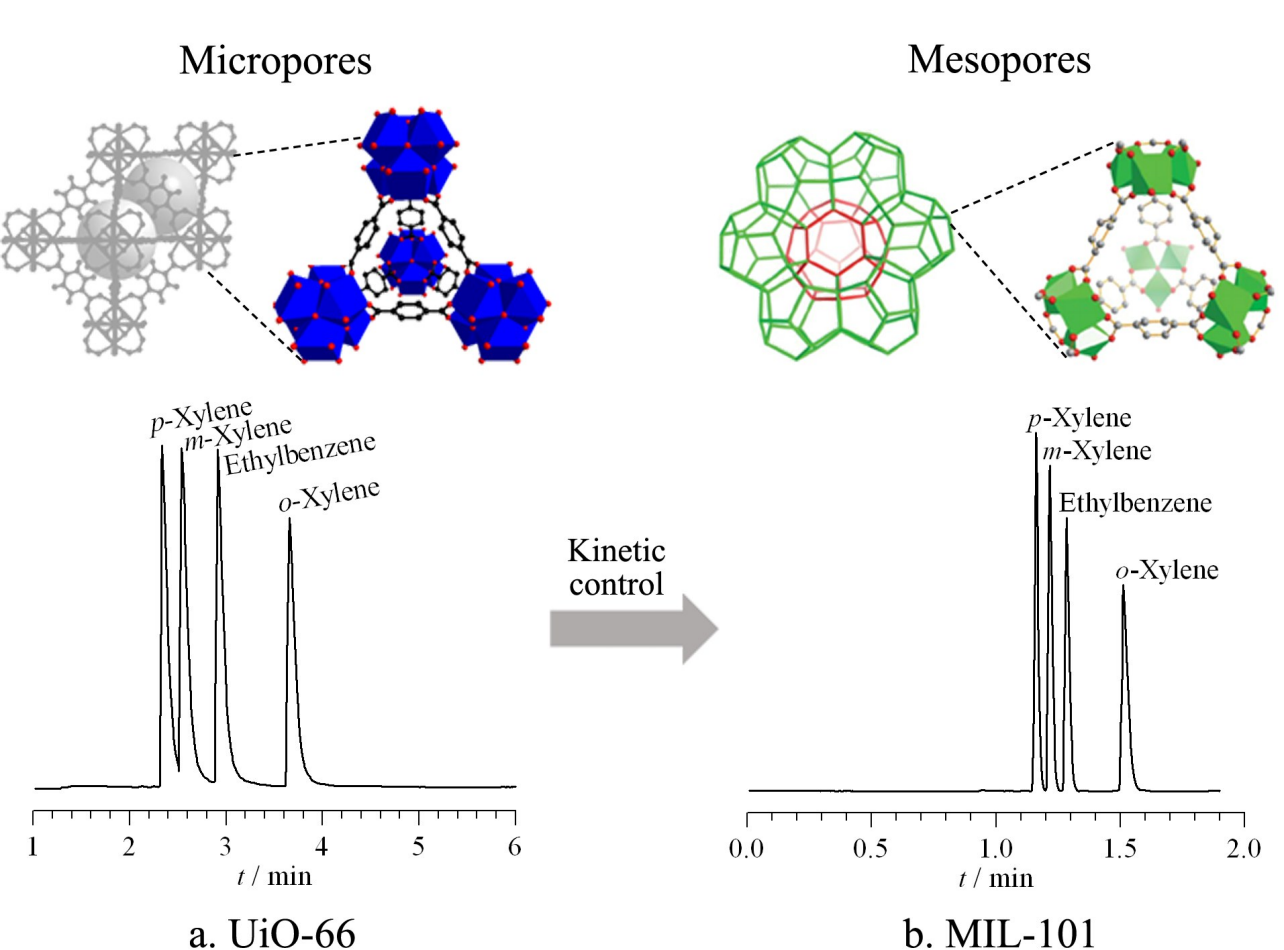
二甲苯和乙基甲苯(a)在UiO-66毛细管柱和(b)在MIL-101毛细管柱中的气相色谱图^[31,32]^

## 2 分离机理

MOFs作为色谱固定相分离异构体可能的分离机理如下:(1)分子筛效应或形状选择性;(2)分析物与固定相内壁之间产生的不同范德华力;(3)不饱和的金属位点与分析物中不同的官能团之间产生的相互作用;(4)*π-π*相互作用、氢键相互作用。

分子筛效应和形状选择性可能是MOFs的主要分离机理。一般情况下,具有支链或较大分子尺寸结构的分子难以进入固定相孔道内,与固定相产生较弱的相互作用,通常先从色谱柱中流出。与之相反,小分子分析物可以有序地进入固定相孔道内,不同分析物主要依靠与MOFs产生不同强弱的范德华力进行分离。同一类型化合物的相对分子质量越大,沸点越高,与固定相之间的相互作用越强,出峰越晚。

另外,进入孔隙中的分析物可能与MOFs特殊的不饱和金属位点相互作用实现分离。例如,UiO-66分离二甲苯和乙基甲苯时主要依赖于它的酸性金属位点。与其他C_8_异构体相比,极性较大的邻二甲苯与UiO-66的酸性位点能产生更强的氢键,使其在UiO-66中的保留时间更长。酸性金属位点能对不同极性的分析物表现出不同的吸附选择性。

MOFs的手性识别能力可能主要依赖外消旋体与手性MOFs中手性活性位点之间的相互作用。此外,手性MOFs和外消旋体之间的氢键和*π-π*相互作用也可能有助于手性识别,实现手性分析物的分离。简而言之,MOFs的手性识别可能是多种影响因素共同作用的结果。

## 3 MOFs气相色谱固定相分离目标物的种类

MOFs气相色谱柱在烷烃、烯烃、二甲苯、外消旋体、含氧有机物和各类有机污染物的分离中发挥着重要作用,能有效提高异构体的分离选择性,具有很好的发展前景。

### 3.1 烷烃

烷烃是重要的石油化工原料,从烷烃混合物中分离出线性烷烃有利于进一步提高烷烃燃料的辛烷值,获得高效燃烧的燃料^[34]^。然而,传统的吸附剂分子筛的种类和数量有限,严重阻碍了烷烃异构体的分离。MOFs具有独特的多孔结构,良好的分子筛效应且能与不同烷烃之间产生不同的范德华作用力,是用于分离烷烃的优异气相色谱固定相材料。因此,本文在表1中总结了MOFs和MOFs复合材料作为气相色谱固定相分离烷烃混合物的例子。

**表 1:** MOFs和MOFs复合材料作为气相色谱固定相分离烷烃异构体

Stationary phase	Formula	Surface area/ (m^2^/g)	Thermal stability/ ℃	Column	Ref.
UiO-66	Zr_6_O_4_(OH)_4_(BDC)_6_	614	500	capillary	[31]
MOF-508	Zn(BDC)(4,4'-Bipy)_0.5_	946	360	packed	[35]
ZIF-8	Zn(mim)_2_	1504	380-500	packed	[36]
ZIF-8	Zn(mim)_2_	1504	380-500	capillary	[37,38]
Graphene-ZIF-8 composite			400	capillary	[39]
UiO-66	Zr_6_O_4_(OH)_4_(BDC)_6_	614	500	packed	[40]
MOF-CJ3	[HZn_3_(OH)(TBC)_2_(2H_2_O)(DMF)]·H_2_O	525	250	capillary	[41]
ZIF-90	Zn(C_4_H_3_N_2_O)_2_	1270	300	capillary	[42]

BDC: 1,4-benzenedicarboxylic acid; 4,4'-Bipy: 4,4'-bipyridine; mim: 2-methylimidazole; TBC: 1,3,5-benzenetricarboxylate; C_4_H_3_N_2_O: imidazolate-2-carboxyaldehyde.

Chen等^[35]^在2006年制备了第一根MOF-508([Zn(BDC)(4,4'-Bipy)_0.5_])填充的气相色谱柱,用于分离烷烃异构体。MOF-508是具有一维纳米孔道(0.4 nm×0.4 nm),并且拥有较大比表面积(946 m^2^/g)和较高热稳定性(360 ℃)的多孔骨架材料,可以有效地分离线性和支链烷烃(见图3)。与线性烷烃相比,支链烷烃具有更大的分子尺寸,更容易接近MOF-508孔道内壁,与MOFs固定相之间产生较强的相互作用。在线性烷烃中,烷烃链长度是决定线性烷烃保留时间的主要因素。通常,烷烃链长度越长,与MOFs固定相之间的范德华相互作用越强,保留时间越长。总的来说,MOFs对烷烃异构体的分离可能主要依赖于烷烃分子形状和尺寸与MOF-508之间的匹配,与MOF-508孔内壁产生不同的范德华力力,进而达到分离。

**图 3:**
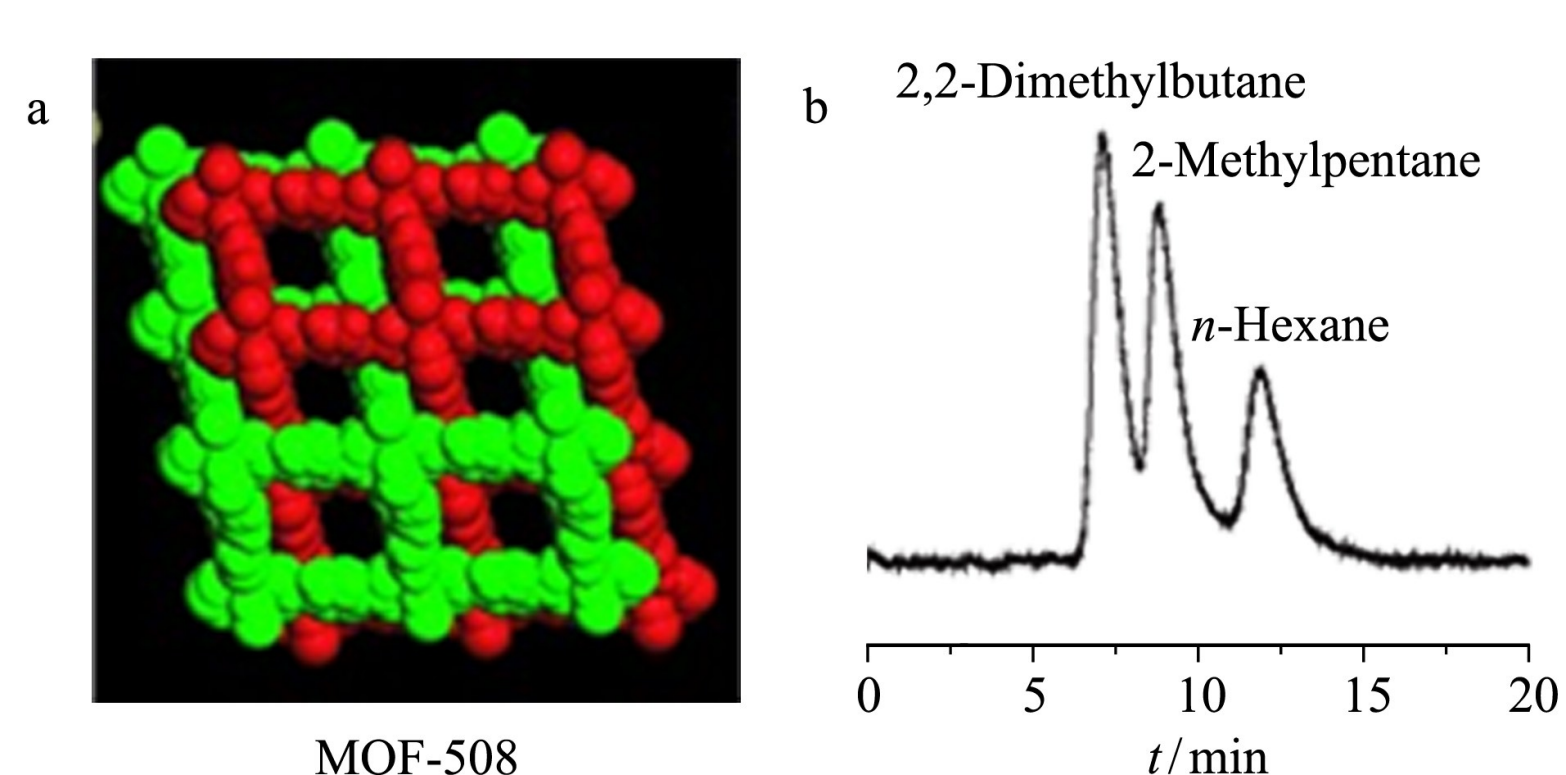
(a)MOF-508结构图和(b)MOF-508填充柱分离烷烃异构体的色谱图^[35]^

ZIF-8(Zn(2-mim)_2_)是一种重要的色谱固定相材料,它具有独特的三维孔道结构,大孔尺寸为1.14 nm,孔窗口尺寸为0.34 nm,比表面积可达1947 m^2^/g。Luebbers等^[36]^以ZIF-8制成了色谱填充柱,用于烷烃异构体分离。Chang等^[37]^采用ZIF-8涂覆毛细管色谱柱,制成了首根具有MOFs尺寸排阻效应的气相色谱柱。该色谱柱对线性烷烃展现出较高的选择性和分离度,能有效从支链烷烃中筛选出线性烷烃。他们还利用ZIF-8-纤维棒固相微萃取与ZIF-8毛细管色谱柱串联^[38]^,成功地对石油燃料和人体血清等实际样品进行了线性烷烃的定量分析。随后,Yang等^[39]^直接将ZIF-8沉积在三维石墨烯上,制得了石墨烯-ZIF-8复合材料。与独立的ZIF-8相比,石墨烯-ZIF-8球材料展现出优异的异构体分离能力,它不仅可以分离烷烃和取代苯异构体,还可以分离顺/反同分异构体。上述研究表明,ZIF-8对烷烃的分离主要依赖于分子筛效应和固定相与目标物烷烃之间的范德华力。根据材料的分子筛效应分析,分子尺寸较大的支链烷烃难以进入MOFs孔内,易于解吸,色谱出峰时间早。而对于容易进入MOFs孔内的线性烷烃而言,烷烃链长度越长,与MOFs固定相之间的范德华力越强,保留时间越长。因此线性烷烃会根据与固定相范德华力的强弱依次解吸并得以分离。这两个因素不仅能区分不同链长的线性烷烃,而且能高效地分离支链和线性烷烃。

ZIF-8分离烷烃异构体时展现出明显的形状选择性,使线性烷烃在固定相中具有更强的保留效果。然而,Bárcia等^[43]^却发现UiO-66(Zr_6_O_4_(OH)_4_(BDC)_6_)中存在独特的反向形状选择性。UiO-66是由十二配位的ZrO_6_(OH)_2_金属簇和对苯二甲酸配体自组装形成的规则有序材料,存在两种不同尺寸的三维刚性孔结构,大孔尺寸为1.1 nm,小孔尺寸为0.8 nm。Chang等^[31]^最早研究了UiO-66在气相色谱分离中的反向形状选择性,并讨论了反向形状选择性对烷烃和芳香族化合物分离的影响(见图2)。与线性烷烃相比,支链烷烃与MOFs之间具有更强的相互作用。上述研究表明,UiO-66的反向形状选择性主要是因为线性和支链烷烃在分离过程中都能进入UiO-66孔内,支链分子侧链上的甲基更容易与孔内壁接触,从而产生更强的范德华力,而线性烷烃反而容易通过MOFs的孔隙,先从色谱柱中流出,展现出反向形状选择性。除此之外,Bozbiyik等^[40]^报道了正己烷和环己烷在UiO-66的反向气相色谱分离和穿透实验。实验表明,环己烷在色谱柱中的保留时间比正己烷更长,因为环己烷尺寸更大,与UiO-66的相互作用更强,仍然符合UiO-66的反向形状选择性。

Fang等^[41]^报道的MOF-CJ3([HZn_3_(OH)(TBC)_2_(2H_2_O)(DMF)]H_2_O)具有525 m^2^/g的比表面积,热稳定性达到250 ℃,能够作为气相色谱固定相分离烷烃异构体。MOF-CJ3中规则的一维管状孔道(1.16 nm×1.16 nm)使线性烷烃和支链烷烃均能通过。因此,烷烃的分离主要依赖于客体分子与疏水MOF-CJ3之间产生的不同范德华力。Wu等^[42]^提出了一种新的方法,制备了ZIF-90(Zn(C_4_H_3_N_2_O)_2_)键合毛细管色谱柱用于气相色谱分离。ZIF-90键合毛细管色谱柱的制备主要是通过Zn^2+^和羧基配位作用,使ZIF-90直接生长在毛细管内壁。该色谱柱可以成功分离烷烃异构体和苯及苯的同系物。

手性气相毛细管色谱柱也能分离烷烃异构体。例如,Xie等制备的[Cu(sala)]_n_^[44]^、Co-(D-Cam)_1/2_(bdc)_1/2_(tmdpy)^[45]^、InH(D-C_10_H_14_O_4_)^[46]^、Ni(D-Cam)(H_2_O)_2_^[47]^、[(CH_3_)_2_NH_2_[Cd(bpdc)_1.5_]·2DMA^[48]^和Zhang等^[49]^制备的Zn(ISN)_2_·2H_2_O手性毛细管色谱柱,能够基线分离线性烷烃(*n*-C_10_至*n*-C_15_)。手性MOFs分离线性烷烃主要依赖于客体分子与固定相的形状和尺寸匹配,进而产生不同的范德华力。

### 3.2 二甲苯异构体和乙基苯

二甲苯异构体包括邻二甲苯(*o*X)、间二甲苯(*m*X)和对二甲苯(*p*X),二甲苯异构体和乙基苯(EB)是重要的化工原料^[50]^,其中对二甲苯是聚酯工业的主要原料,邻二甲苯可用于生产塑化剂,间二甲苯用于生产异酞酸,乙基苯常用于生产苯乙烯。此外,二甲苯异构体和乙基苯也是环境监测中的重要目标物,因此对其高效分离十分迫切。但由于二甲苯异构体和乙基苯的沸点相近、分子尺寸相似,4个异构体的高效分离是分离科学中的一大挑战,被Nature杂志评述为影响世界的7种化学分离之一^[51]^。近年来,MOFs在分离二甲苯和乙基苯异构体方面取得了较大进展。本文在表2中总结了MOFs和MOFs复合材料作为气相色谱固定相分离二甲苯异构体和乙基苯的例子。

**表 2:** MOFs和MOFs复合材料作为气相色谱固定相分离二甲苯和乙基甲苯异构体

Stationary phases	Formula	Surface area/(m^2^/g)	Thermal stability/ ℃	Column	Ref.		
MIL-101	Cr_3_O(H_2_O)_2_F(BDC)_3_	2376-2907	300	capillary			[32]
Zr-BTB	[Zr_6_O_4_(OH)_4_(BTB)_2_](H_2_O)_4_(OH)_4_(FA)_0.5_	338.3	400	capillary			[33]
MIL-47	V^IV^O(BDC)	800	350	packed			[52]
MOF-5	Zn_4_O(BDC)_3_		500	packed			[53]
MCF-50	[Zn(Hpidba)]·2.6DMF H_2_O	1319	350	capillary			[54]
MAF-6	RHO-[Zn(eim)_2_]	1695	400	capillary			[55]
ZIF-8@PDMS core-shell microspheres		1290	500	packed			[56]

BTB: 1,3,5-(4-carboxylphenyl)-benzene; FA: formic acid; Hpidba: 4,4-(2-(pyridin-2-yl)-1*H*-imidazole-4,5-diyl)dibenzoic acid; eim: 2-ethylimidazole.

Gu等^[53]^制备两根Zn-MOF色谱填充柱(包括MOF-5和单斜MOF),用于二甲苯和乙基苯异构体的吸附和分离。由于两种Zn-MOF的比表面积、极性、孔窗尺寸和结构存在很大差异,因此他们对异构体的选择性和分离效果不同。MOF-5(Zn_4_O(BDC)_3_)和单斜MOF的比表面积分别为773 m^2^/g和225 m^2^/g,导致MOF-5的吸附效果明显优于单斜MOF。通过测定固定相的麦式常数,确定MOF-5为非极性固定相,而单斜MOF为中等极性固定相,非极性的MOF-5理论塔板数(267块/m)比中等极性单斜MOF(76块/m)更高。在MOF-5中,其孔尺寸约为1.2 nm,4个异构体具有相似的动力学直径(0.585~0.685 nm),在分离过程中不存在尺寸障碍,具有相似的扩散系数。因此范德华力是其分离的主要因素,4种异构体按照沸点顺序先后出峰。而单斜MOF中,MOFs孔形状起主要作用,p*X*在其三角孔中的吸附效果优于其他异构体,因此最后出峰。Finsy等^[52]^合成具有三维孔道的MIL-47(V^IV^O(BDC)),制备了MOFs气相色谱填充柱分离二甲苯和乙基苯异构体,吸附分离效果受压力和温度的影响较大。

Gu等^[32]^在2010年报道了首根MOFs毛细管气相色谱柱用于分离二甲苯异构体和乙基苯。MIL-101(Cr_3_O(H_2_O)_2_F(BDC)_3_)具有0.8 nm的微孔和2.9 nm、3.4 nm的介孔、开放的金属位点和良好的化学与热稳定性。MIL-101毛细管柱理论塔板数达3800(块/m),在恒温条件下100 s内能基线分离二甲苯异构体和乙基苯,优于商品化柱和已报道的其他MOFs色谱。值得注意的是,MIL-101毛细管柱对4个异构体具有独特的选择性,流出顺序为*p*X<*m*X<EB<*o*X,不同于常规的沸点顺序(EB<*p*X<*m*X<*o*X)。这源于MOFs与分析物之间独特的主-客体相互作用,还归因于MIL-101的适宜极性和开放的金属位点。

最近,Tao等^[33]^合成了具有超薄多孔特性的二维Zr-BTB纳米片([Zr_6_O_4_(OH)_4_(BTB)_2_](H_2_O)_4_(OH)_4_(FA)_0.5_),其作为色谱固定相有效提高了分析物在分离过程中的扩散效率,实现了二甲苯异构体和乙基苯的分离。此外,制备的Zr-BTB-FA毛细管柱能够基线分离其他5组二取代苯异构体(见图4)。此MOF纳米片对6组异构体均展现出独特的对位选择性,其中间位和对位异构体之间的分离度远远高于商用柱(HP-5MS和VF-MAX)和其他三维MOFs毛细管柱。研究表明,取代苯异构体的分离主要依赖于二维纳米片规整堆叠形成的有序纳米孔道。有序孔道的形成机理是在干燥过程中,乙醇溶剂辅助相邻层间脱去乙醚形成Zr-O-Zr键,诱导纳米片形成规整的亚纳米孔道,实现了取代苯异构体的高效分离。

**图 4:**
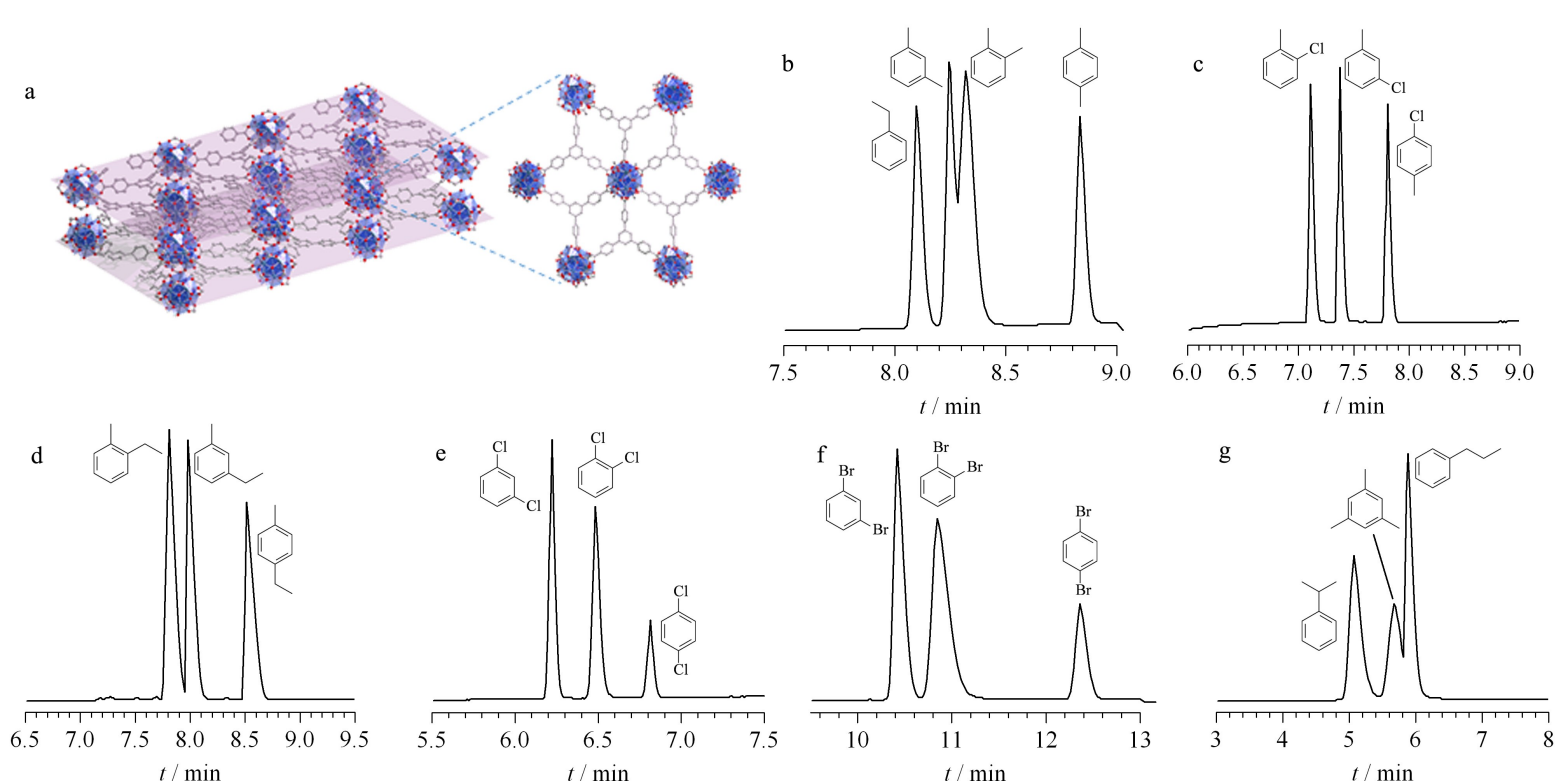
(a)二维Zr-BTB-FA纳米片的结构图和(b~g)2D-Zr-BTB-FA色谱柱分离6组苯取代物异构体的色谱图^[33]^

Lin等^[54]^合成的MCF-50([Zn(Hpidba)] 2.6DMF·H_2_O)不仅有一维的大孔结构,而且有相互连通的柔性小孔通道。制备的MCF-50毛细管柱对二甲苯异构体具有高效选择性,它们流出顺序为*o*X<*m*X<*p*X。随后,He等^[55]^制备的MAF-6(RHO-[Zn(eim)_2_])柱在3 min内实现了二甲苯异构体的基线分离,流出顺序为*m*X<*p*X<*o*X。

### 3.3 外消旋体

手性是生物系统的基本特征,如核酸、蛋白质、多糖都具有手性^[57]^。不同对映体通常具有不同的药物活性、代谢途径和毒理作用。因此,对映体的拆分在立体化学、生物样品制备和制药工业中至关重要。近年来,各种手性MOFs被广泛应用于气相色谱中分离外消旋体。表3总结了手性MOFs作为固定相分离外消旋体的例子。

**表 3:** MOFs和MOFs复合材料作为气相色谱固定相分离二甲苯和乙基甲苯异构体

Chiral stationary phases	Surface area/(m^2^/g)	Thermal stability/ ℃	Racemates	Ref.
[Cu(sala)]_n_	11	220	1, 2, 3, 4, 5, 6, 7, 8, 9, 10, 11	[44]
Co(D-Cam)_1/2_(bdc)_1/2_(tmdpy)		250	1, 7, 9, 11, 12, 13, 14	[45]
InH(D-C_10_H_14_O_4_)_2_	497	230	1, 7, 9, 11, 14, 12, 15	[46]
Ni(D-Cam)(H_2_O)_2_		250	1, 3, 4, 7, 9, 10, 13, 14, 15	[47]
[(CH_3_)_2_NH_2_][Cd(bpdc)_1.5_]·2DMA	43	350	1, 4, 13, 15, 16, 17	[48]
Zn(ISN)_2_·2H_2_O	150	350	1, 3, 7, 9, 10, 11	[49]
In_3_O(obb)_3_(HCO_2_)(H_2_O)		350	4, 7, 9, 12, 17	[58]
[Zn_2_(D-Cam)_2_(4,4'-bpy)]_n_		400	1, 9, 12, 14, 17, 21,	[59]
Co(D-Cam)_1/2_(bdc)_1/2_(tmdpy)+β-CD		250	1, 4, 12, 18, 19, 20, 22, 25, 43	[60]
InH(D-C_10_H_14_O_4_)_2_+β-CD			1, 4, 11, 12, 18, 19, 20	[61]
[Cd(LTP)_2_]_n_+β-CD		220	1, 3, 8, 11, 12, 14, 19, 20, 22, 23, 24, 25, 26, 27	[62]
MIL-101(Al)-NH_2_-Xs	441-1292	250-350	1, 14, 23, 36, 37, 38, 39, 40, 41, 42	[63]

Sala: *N*-(2-hydroxybenzyl)-L-alanine; D-cam and D-C_10_H_14_O_4_: D-camphoric acid; ISN: isonicotinate; bdc: 1,4-benzenedicarboxylate; tmdpy: 4,4'-trimethylenedipyridine; bpdc: 4,4'-biphenyldicarboxylate; obb: 4,4'-oxybis(benzoic acid); *β*-CD: *β*-cyclodextrin; bpy: 4,4'-bipyridine; LTP: L(-)-thiazolidine-4-carboxylic acid; Xs: *S*-2-phe-nylpropionic acid, *R*-1,2-epoxyethylbenzene, (+)-diacetyl-L-tar-taric anhydride, L-proline and 1*S*-(+)-10-camphorsulfonyl chloride. The name of racemates: 1. citronellal; 2. camphor; 3. alanine; 4. leucine; 5. leucine; 6. isoleucine; 7. proline; 8. 2-methyl-1- butanol; 9. 1-phenyl-1,2-ethandiol; 10. phenyl-succinic acid; 11. 1-phenyl-ethanol; 12. limonene; 13. glutamic acid; 14. 2-amino-1-butanol; 15. methionine; 16. threonine; 17. aspartic acid; 18. methyl-L-*β*-hydroxyisobutyrate; 19. dihydrocarvyl acetate; 20. menthol; 21. mandelic acid; 22. 2-hexanol; 23. 1-phenylethyl amine; 24. 1-cyclohexyl amine; 25. rose oxide; 26. 2-phenyl-1-propanol; 27. arginine; 28. 1-phenyl-2-propanol; 29. *α*-vinylbenzyl alcohol; 30. 2-phenylpropanenitrile; 31. 1-phenyl-1-butanol; 32. 1-phenyl-1-pentanol; 33. 1-phenyl-2-butanol; 34. *α*-cyclopropylbenzyl alcohol; 35. 2-phenylbutyronitrile; 36. 2-methyl-2,4-pentanediol;37. 1,2-pentanediol, 38. 2-butanol; 39. 1-heptyn-3-ol; 40. 1-amino-2-propanol; 41. mandelonitrile; 42. methyl-2-chloropropionate; 43. linalool.

Xie等^[44]^首次将手性MOFs用作固定相进行气相色谱分离外消旋体。他们通过动态涂覆的方法制备了具有三维手性孔道结构的[Cu(sala)]*_n_*毛细管色谱柱,实现了多种氨基酸衍生物的拆分(见图5)。[Cu(sala)]*_n_*手性柱在分离D/L构型的外消旋体(异亮氨酸、亮氨酸、苯基琥珀酸、缬氨酸、丙氨酸和脯氨酸)时,L型对映体总是在D型对映体之后出峰,说明MOFs的手性孔道微环境与L构型的对映体匹配度更高,二者之间具有更强的相互作用。

**图 5:**
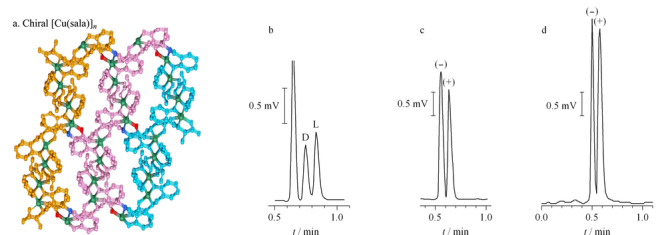
(a)手性[Cu(sala)]*_n_*的三维结构图和[Cu(sala)]*_n_*手性柱分离(b)异亮氨酸、(c)香茅醛、(d)1-苯基-1,2-乙二醇外消旋体的气相色谱图^[44]^

随后,其他一些手性MOFs包括Co(D-Cam)_1/2_(bdc)_1/2_(tmdpy)、InH(D-C_10_H_14_O_4_)_2_、[(CH_3_)_2_NH_2_][Cd(bpdc)_1.5_]·2DMA、In_3_O(obb)_3_(HCO_2_)(H_2_O)和Ni(D-Cam)(H_2_O)_2_也被成功合成并用于气相色谱分离外消旋体^[45-48,58]^。他们利用外消旋体、同分异构体、烷烃、醇类和Grob试剂对色谱柱的分离性能进行了测试。结果显示,这些手性MOFs具有高效分离香茅醛、樟脑、丙氨酸、亮氨酸、缬氨酸、异亮氨酸、1-苯基-1,2-乙醇、苯基琥珀酸和1-苯基-乙醇等外消旋体的能力,展现出对外消旋体良好的选择性和识别能力。除此之外,Xue等^[59]^合成了大孔三维手性MOF([Zn_2_(D-Cam)_2_(4,4-bpy)]*_n_*),该手性色谱柱不仅实现了外消旋体的高效分离,而且还能快速分离*α*,*β*-紫罗酮和顺、反柠檬醛异构体。上述研究表明,手性MOFs展现出的优异分离性能主要依赖于它们的手性孔道,不同形态的手性分子与MOFs之间产生不同的相互作用,展现出对映体选择性。此外,氢键和范德华力也有利于提高气相色谱中的手性识别能力和对映体选择性。

据报道,环糊精及其衍生物是一类具有高效分离能力的气相色谱固定相^[64]^。Liu等^[60]^首次将*β*-环糊精和手性MOFs(Co(D-Cam)_1/2_(bdc)_1/2_(tmdpy))结合制成手性MOFs-*β*-CD复合材料用于气相色谱分离外消旋体。随后,Yang等^[61,62]^分别用两种手性InH(D-C_10_H_14_O_4_)_2_和[Cd(LTP)_2_]*_n_*与*β*-CD结合制得两根MOF-*β*-CD毛细管柱。在气相色谱分离实验中,与A柱(手性MOF柱)和B柱(氯化钠+*β*-CD柱)相比,C柱(手性MOF+*β*-CD柱)对外消旋体具有更好的分离能力。由此可以看出,*β*-环糊精和手性MOFs的协同作用可以有效提高其对对映体的分辨能力和选择性。

最近,Kou等^[63]^报道了一种简单的后修饰合成手性MOFs的方法。他们选择具有孔道结构和氨基的MOF(MIL-101(Al)-NH_2_)为进行化学后修饰的反应基体。其在MIL-101(Al)-NH_2_中分别后修饰了5种不同的手性基团,得到5种具有不同手性位点的手性MOFs(见图6),并制备5根手性MOFs毛细管柱用于外消旋体分离。结果表明,修饰的手性基团不同,MOFs孔道内的手性环境不同,进而对分析物产生不同的分离选择性。此研究有利于我们定向合成与外消旋体特异性相互作用的手性MOFs,为新型手性MOFs的设计和合成提供了广阔的应用前景。

**图 6:**
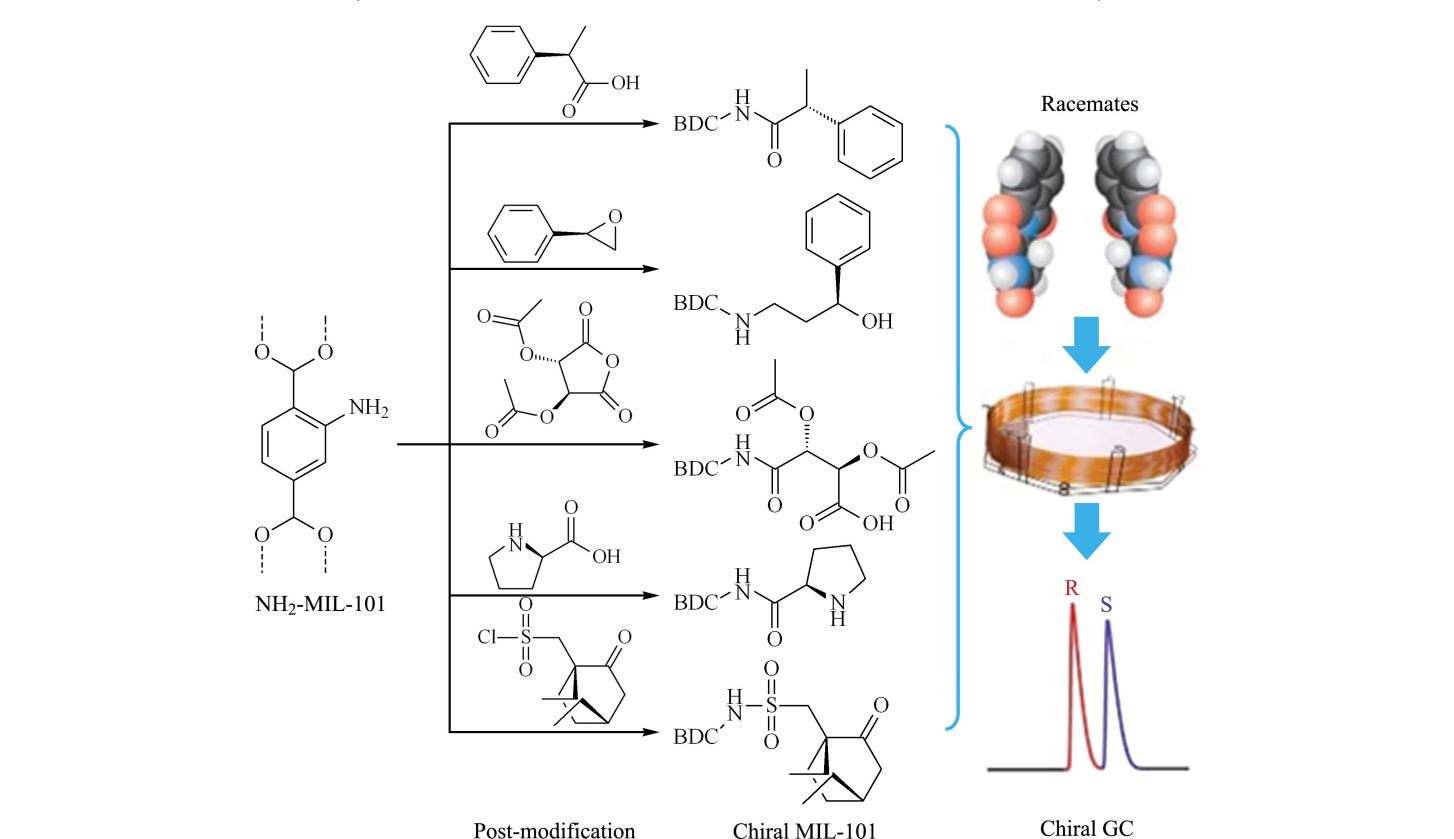
手性MIL-101(Al)-Xs的后合成示意图^[63]^

### 3.4 含氧有机物

Zhang等^[65]^合成具有四重互穿金刚石拓扑结构的MOF([Ni(pybz)_2_]),将其用于气相色谱分离含氧有机物。分离的含氧有机物包括6组含有不同官能团的同系物(R-OH、H-(R)C=O、(RR')C=O、HO-(R)C=O、RO-C(R')=O、R-O-R')。该毛细管柱对薄荷酮、乙酸甲酯和丁香酚等18种不同官能团的化合物具有良好分离效果。此外,Zhang等^[66]^合成另一种四重互穿的MOF(Cd(D-Cam)(tmdpy)),用于芳香烃衍生物(醚、酸和醇)和线性烷烃衍生物(醛、酸和醚)的气相色谱分离。MOFs与分析物之间存在主-客体相互作用,使得固定相具有良好的分离效果。

随后,Zheng等^[67,68]^合成的(Zn_2_(bdc)(L-lac))和([Mn_3_(HCOO)_2_(D-cam)_2_(DMF)_2_]*_n_*)用于分离3类含氧有机物,包括碳氢化合物衍生物(醛、醇、酯)、双苯基芳香烃衍生物(醚)和芳香烃衍生物(氨基酸酯和二酯)。Münch等^[69]^采用循环技术在毛细管柱内壁涂覆了HKUST-1(Cu_3_(BTC)_2_)。HKUST-1开放的金属位点与具有不同电子云密度的化合物之间产生不同的相互作用,进而实现了二乙醚、四氢呋喃、二异丙醚等含氧有机物的分离。

Xie等^[44,45,46,47,48]^合成的手性MOFs材料也被用于分离含氧有机物。例如[Cu(sala)]*_n_*、Co(D-Cam)_1/2_(bdc)_1/2_(tmdpy)、InH(D-C_10_H_14_O_4_)_2_、[(CH_3_)_2_NH_2_][Cd(bpdc)_1.5_]·2DMA和Ni(D-cam)(H_2_O)_2_ 5种手性MOFs在分离线性醇类和紫罗酮时展现出较高的选择性和分离度。除此之外,[(CH_3_)_2_NH_2_][Cd(bpdc)_1.5_]·2DMA还能分离顺式和反式柠檬醛。一般来说,含氧有机物的分离主要依赖于分析物与MOFs之间不同强弱的范德华力。

### 3.5 有机污染物

近年来,挥发性有机污染物(VOCs)、多氯联苯(PCBs)、多环芳烃(PAHs)、多溴联苯醚(PBDEs)、六氯环己烷(HCHs)等有机污染物引起了人们的广泛关注^[70]^。这类有机污染物的同分异构体具有相似的物理化学性质,难以被分离。MOFs作为一种新型的气相色谱固定相,在分离有机污染物方面也有相关报道。在表4中总结了MOFs和MOFs复合材料作为气相色谱固定相用于分离含氧有机物和有机污染物的例子。

**表 4:** MOFs和MOFs复合材料分离含氧有机物和有机污染物

Stationary phase	Surface area/(m^2^/g)	Thermal stability/ ℃	Analyte	Ref.
Ni(pybz)_2_	228	220	oxy-organic	[65]
Cd(D-Cam)(tmdpy)		215	oxy-organics	[66]
Zn_2_(bdc)(L-lac)	190	350	oxy-organics	[67]
[Mn_3_(HCOO)_2_(D-cam)_2_(DMF)_2_]_n_		230	oxy-organics	[68]
HKUST-1 (Cu_3_(BTC)_2_)	404-629	220-280	oxy-organics	[69]
IRMOF-1 (Zn_4_O(BDC)_3_)	2517	400	organic pollutants	[71]
IRMOF-3 (Zn_4_O(NH_2_-BDC)_3_)	1957	320	organic pollutants	[71]
IRMOF-8 (Zn_4_O(NDC)_3_)	1343	500	organic pollutants	[72]
184 silicone@MAF5		400	organic pollutants	[73]

pybz: 4-(4-pyridyl) benzoic acid; D-Cam: D-(+)-camphoric acid; tmdpy: 4,4'-trimethylenedipyridine; bdc: 1,4-benzenedicarboxylate; BTC: 1,3,5-benzenetricarboxylic acid; NDC: naphthalene-2,6-dicarboxylate; L-lac: L-lactate.

Gu等^[71]^合成了IRMOF-1(Zn_4_O(BDC)_3_)和IRMOF-3(Zn_4_O(NH_2_-BDC)_3_)分离有机污染物PCBs(见图7)。IRMOF柱具有再现性好、分辨率高、选择性高和线性范围广的特点。PCBs异构体的快速分离主要依赖于其与IRMOF孔尺寸(1.12 nm和0.96 nm)的相互匹配。IRMOF-1和IRMOF-3柱的理论塔板数分别为2293块/m和2063块/m。此外,六氯联苯在IRMOF-3色谱柱中的分离选择性比IRMOF-1更好,是因为IRMOF-3中存在氨基。Gutiérrez等^[72]^利用反向气相色谱技术研究了3种不同IRMOF对30多种VOCs的吸附效果。通过测定VOCs在MOFs的吸附焓和平衡常数,发现IRMOF-1骨架结构中缺陷越多,与被吸附物之间产生的相互作用力越强。然而,VOCs与MOFs之间的相互作用机理鲜有报道,需要进行进一步基础实验研究。

**图 7:**
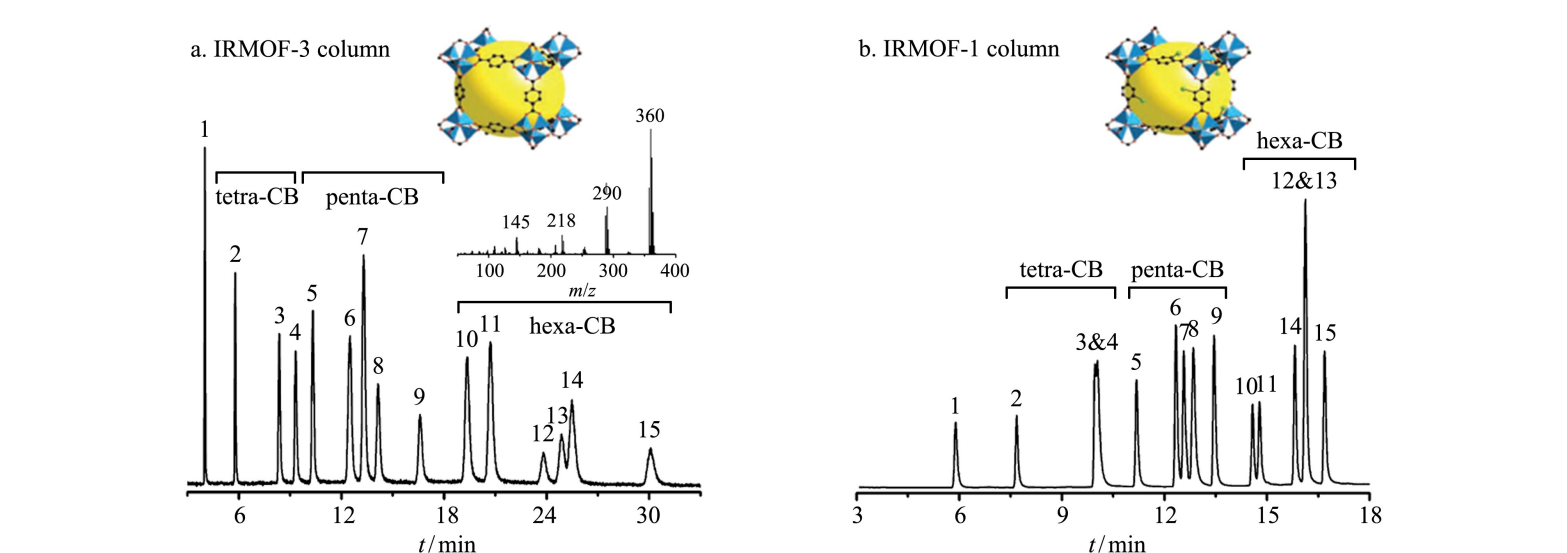
(a) IRMOF-3柱和(b) IRMOF-1柱分离多氯联苯的色谱图^[71]^

MAF-5(Zn(eim)_2_)具有不同大小的环形孔结构,其中Zn_6_(eim)_6_环的直径为0.22~0.49 nm, Zn_8_(eim)_8_孔的长度为0.4 nm,宽度为0.58~0.90 nm。这使它在色谱分离领域展现出优异的分离性能。Tian等^[73]^提出了一种新的制备毛细管的方法,首先在毛细管内壁镀上184硅橡胶作为保护薄膜,然后再将MAF-5涂覆在其内壁。制备的毛细管色谱柱具有坚固、稳定的184硅橡胶@MAF-5固定相涂层,能够分别在10 min和14 min以内成功分离多环芳烃异构体和有机氯农药。与IRMOF柱和HP-5MS柱相比,这种184硅橡胶@MAF-5柱分离时间更短,但目标物的出峰顺序没有发生改变,表明这3根柱子的分离机理相似。

## 4 总结与展望

综上所述,我们总结了MOFs固定相分离不同种类目标物的例子。采用合适的MOFs作为气相色谱固定相可以高效地分离烷烃、二甲苯异构体和乙基苯、外消旋体、含氧有机物和几种持久性有机污染物,这在化学工业和环境科学中具有重要意义。到目前为止,虽然每年都有大量的MOFs被合成,但仍然只有少数的MOFs作为固定相用于气相色谱分离,主要是因为我们无法根据分析物来精准合成相应的MOFs,实现分离。本综述从分析物和MOFs相互作用的角度出发,分析了MOFs的分离机理,从一定程度上为解决这一问题提供了参考。首先,根据分子筛效应,可以通过选择不同的金属中心和不同长度的配体,设计合成不同孔道大小的MOFs,实现对不同大小目标物的有效分离。其次,通过增加MOFs的缺陷,暴露更多的金属不饱和位点,有效分离不同极性的分析物。再者,添加手性基团,合成手性MOFs可以有效提高固定相对外消旋体的手性拆分能力。在上述基础上,通过制备MOFs复合材料,可丰富MOFs固定相的种类,进一步提高对分析物的选择性和分离度。

MOFs作为气相色谱固定相分离分析物还存在一些不足。首先,分离机理的研究不够深入,部分MOFs对分析物的分离主要依赖于分子筛效应和MOFs与分析物之间的范德华力,但是有的MOFs中存在不同尺寸和不同环境的孔道,无法确定不同的孔道在分离中的具体作用。再比如,手性MOFs分离外消旋体的机理尚不清晰,还需要进一步研究。因此,有必要加大对MOFs分离机理的研究,为设计合成分离效果好的MOFs提供更多的理论指导。其次,MOFs的成本高、产量低也是MOFs作为气相色谱固定相亟需克服的困难之一。应筛选更廉价的配体和金属盐,降低MOFs合成的成本,提高MOFs的产量,增加MOFs在实际工业应用上的可行性。最后,应开拓分析目标物,进一步推进MOFs气相色谱柱的商业化应用。
